# Alpha-linolenic acid inhibits hepatocellular carcinoma cell growth through Farnesoid X receptor/β-catenin signaling pathway

**DOI:** 10.1186/s12986-022-00693-1

**Published:** 2022-08-23

**Authors:** Shu Feng, Xingming Xie, Chaochun Chen, Shi Zuo, Xueke Zhao, Haiyang Li

**Affiliations:** 1 Department of Infectious Diseases, The Affiliated Hospital of Guizhou Medical University, 28 Guiyi Road, Guiyang, Guizhou People’s Republic of China; 2 Department of Hepatobiliary Surgery, The Affiliated Hospital of Guizhou Medical University, 28 Guiyi Road, Guiyang, Guizhou People’s Republic of China

**Keywords:** Alpha-linolenic acid, Farnesoid X receptor, β-catenin, Hepatocellular carcinoma

## Abstract

**Background:**

Altered lipid profiles are frequently present in cancer, and it is necessary to elucidate the role of changed lipid profiles in hepatocellular carcinoma (HCC). We conducted this study to investigate the changed lipid profile in HCC tissues and discover some remarkably changed lipid components, and to explore the function of changed lipid components in HCC development.

**Methods:**

Gas chromatography/mass spectrometer (GC/MS analysis) was employed to measure the abundance of fatty acids between HCC tissues and adjacent noncancerous tissues. The proliferative ability of HCC cells was determined by Cell Counting Kit-8 and EdU assays. Transwell and wound healing assays were employed to determine the migratory ability of HCC cells. Protein expression was assessed by western blot assay.

**Results:**

GC/MS analysis revealed that alpha-linolenic acid was present at lower levels in HCC tissues than that in the adjacent noncancerous tissues. Alpha-linolenic acid inhibited the proliferation, migration and invasion of HCC cells in vitro. Western blotting showed that alpha-linolenic acid treatment increased Farnesoid X receptor expression and decreased β-catenin and cyclinD1 expression.

**Conclusions:**

Alpha-linolenic acid suppresses HCC progression through the FXR/Wnt/β-catenin signaling pathway. Rational use of alpha-linolenic acid may prevent the occurrence of liver cancer in the future.

**Supplementary Information:**

The online version contains supplementary material available at 10.1186/s12986-022-00693-1.

## Background

Liver cancer ranks sixth among malignant tumours in terms of morbidity and ranks third mortality of cancer-related death in 2020 in the global [1]. Fatty acid alterations are observed in colon cancer [2], prostate cancer [3], ovarian cancer [4] and HCC [5, 6]. Fatty acids play a crucial role in the cancer progression. In HepG2 cells, DHA suppresses the proliferation and induces apoptosis [7]. Cis9, trans11 conjugated linoleic acid induces HCC cells apoptosis through activation PPAR-γ signaling pathway [8]. In human breast cancer, alpha-linolenic acid blocks the epithelial–mesenchymal transition (EMT) of cancer cells through inactivation the expression of twist1 expression [9]. Oleate (C18:1) promotes cell proliferation through stabilization of β-catenin in renal carcinoma [10]. It is necessary to elucidate the alterations in the in HCC to understand the role of altered fatty acids in HCC development.

In human HCC, dysregulation of Wnt/β-catenin signaling frequently happened [11]. Farnesoid X receptor (FXR), a transcriptional factor encoded by NR1H4, serves as a tumour suppressor in HCC [12]. In HCC and colorectal cancer, it was reported that FXR binds with β-catenin to block the formation of the core β-catenin/TCF4 complex, which promotes cyclinD1 expression for tumour cell proliferation [13, 14]. However, whether or not the altered lipid profile has an effect on the FXR/Wnt/β-catenin signaling in the HCC progression is still unknown.

In this study, the aims are to identify the altered lipid profile in HCC tissues and investigate the effect of fatty acids on proliferation, migration and invasion of HCC cells, and explore the correlation between fatty acids and FXR /Wnt/β-catenin signaling in HCC.

## Methods

### Study design and participants

Ten HCC tissues and adjacent noncancerous tissues were used for the analysis of lipid compositions. HCC tissues and corresponding adjacent noncancerous tissues were used for western blotting to detect the levels of β-catenin and FXR. Patient tissues were obtained from the Affiliated Hospital of Guizhou Medical University between July 2020 and January 2021. Primary HCC was diagnosed. These patients did not receive any treatment before surgery. Patient tissues and their medical records were used in this study with their consent. Ethical permission of this study was obtained from the Ethics Committee of the Affiliated Hospital of Guizhou Medical University (NO. 2019–231).

### GC/MS analysis for fatty acids

Lipid extraction for GC/MS analysis was conducted at LC Bio (Hangzhou, China). Tissues (20 mg) were grinded with 1 ml of cold methanol using TissueLyser at 50 Hz for 90 s. Then, 40 μl homogenate, 10 μl internal standard (0.2 mg/ml C17:0-d33, 0.4 mg/ml BHT) methanol solution and 200 μl methanol-hexane (4:1, v/v) were mixed and subsequently placed in liquid nitrogen for 10 min. After adding 20 μl acetyl chloride, the mixture was kept in the dark for 24 h. Then, the mixture and 0.5 ml of 6% K_2_CO3 solution were cultured in an ice bath to terminate the reaction and neutralize the hydrochloric acid produced in the reaction. Then, 50 μl of N-hexane was added to the mixture and centrifugated at 3000 rpm for 10 min. Finally, the top layer was collected for GC/MS analysis.

All samples were analysed on a GC/MS (7890b-5977b, GC/MS, Agilent, USA). The chromatographic conditions were as follows: chromatographic column, Agilent DB-225 (10 m × 0.1 mm ID × 0.1 µm); temperature of the injection port and detector, 230 °C; and sample injection volume, 1 μL. The mass spectrometry conditions were as follows: sampling mode, SCAN; ion source temperature, 230 °C; and quadrupole temperature, 150 °C. The data acquisition and analysis were conducted in Mass Hunter software (Version B.08.00, Agilent, USA).

### HCC cell lines

HepG2, Huh7 and L02 cells were purchased from the Cell Bank of the Chinese Academy of Sciences (Shanghai, China). DMEM (GibcoBRL, USA) medium was used to culture cell lines at 37 °C with 5% CO_2_.

### Cell Counting Kit-8(CCK-8) assay

The CCK-8(Dojindo) assay was employed to analyse the proliferation. HCC cells (3000/well) were seeded into 96-well plates with 100 µl complete medium (control) or 100 µl different concentrations of alpha-linolenic acid (Sigma–Aldrich, St. Louis, MO, USA). HCC cells (3000/well) were seeded into 96-well plates with 100 µl complete medium (control) or 100 µl different concentrations of GW4064 (the FXR agonist, CAS No.: 278779-30-9, MCE). For the OD value testing, each well was cultured with 10 µl of CCK-8 reagent for 2 h. The OD values were measured at 450 nm with a SYNERGY H4 microplate reader.

### EdU staining

Cells were seeded in 6-well plates containing coverslips. When grown to 60% confluency, cells were cultured with 5-ethynyl-2′-deoxyuridine (EdU-594, Beyotime, China) for 2 h. Phosphate-buffered saline washed the cells three times for 5 min and then cells were fixed with 4% formaldehyde for 10 min. Next, the cells were permeabilized with 0.3% Triton X-100 (Sigma, USA) for 15 min. Then, the cells were incubated with click reaction liquid for 30 min and subsequently incubated with Hoechst 33,342 for 10 min without light. Finally, the coverslips containing cells were photographed by a Zeiss Laser Microscope.

### Cell migration and invasion assays

When cells grow to 100% confluency in 6-well plates, wounds are generated by a 200 µl pipette tip. These photos of wounds serve as the time point of 0-h. Cells were cultured with or without alpha-linolenic acid in free serum medium for 48 h, and the photos of wounds were collected. For the Transwell assay, the cells were seeded with 2 × 10^4^ cells/well into the upper compartment without serum in Transwell chambers (8 µm pore size, Corning Incorporated). The upper compartments precoated with Matrigel are used for invasion assay, and upper compartments for the migration assay are without Matrigel. And the lower compartment contains 10% foetal bovine serum with 200 µM alpha-linolenic acid (700 µl). The cells are fixed with 4% paraformaldehyde and stained with 0.1% crystal violet after incubation for 48 h. Cells from the upper compartment were wiped away with cotton swabs. An inverted microscope is used to photograph the migrated cells.

### Western blotting

Total proteins were extracted with RIPA buffer containing protease inhibitor, and the protein concentration was tested by a BCA kit (Solarbio, Beijing, China). Proteins were separated by 10% SDS-PAGE and transferred to a nitrocellulose membrane. 5% milk blocked the membrane for 1 h at room temperature, and then primary antibody was used to interact with target protein at 4 °C overnight. Then second antibody interacted with primary antibody for 1 h at room temperature. The primary antibodies included anti-β-catenin (1:9000, 51,067-2-AP, Proteintech, China), anti-NR1H4 (1:2000, 25,055-1-AP, Proteintech, China), anti-GAPDH (1:7000, 10,494-1-AP, Proteintech, China), anti-cyclin D1 (1:1000, 26,939-1-AP, Proteintech, China) and anti-c-myc(1:8000, 10,828-1-AP, Proteintech, China). The secondary antibody was goat anti-rabbit IgG (1:6000, SA00001-2, Proteintech, China).

### Statistical analysis

Normality of distribution was evaluated by the Kolmogorov–Smirnov test. Student’s t test was performed based on the normally distributed continuous data. The data were not normally distributed, so the Mann–Whitney U test was adopted. Statistical analysis and graph production were performed with GraphPad Prism 8 (GraphPad Software, USA). A *p* value less than 0.05 was served as statistical difference.

## Results

### GC/MS-based lipidomic analysis in human HCC tissues

**Fig. 1:**
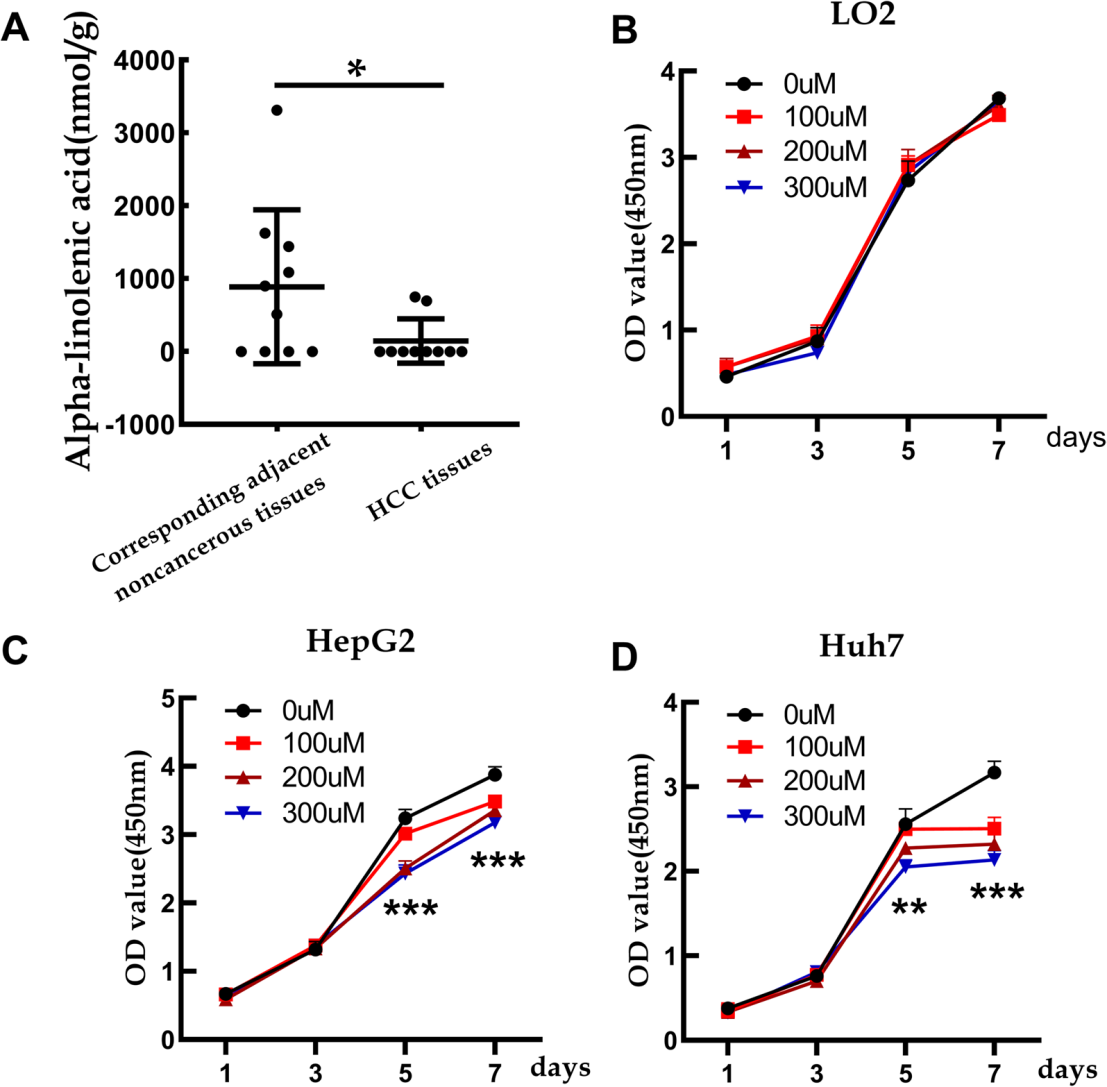
Alpha-linolenic acid inhibits HCC cell proliferation, as tested by CCK-8 assay. **A** The content of alpha-linolenic acid in HCC tissues and the adjacent noncancerous tissues through GC/MS analysis. **B** CCK8 assay for proliferation in the normal liver cell line L02. **C** The CCK8 assay for proliferation in HepG2 cells. **D** The CCK8 assay for proliferation in Huh7 cells. *p < 0.05, **p < 0.01, ***p < 0.001

GC/MS analysis was used to assess the abundance of fatty acids in HCC tissues and adjacent noncancerous tissues. Alpha-linolenic acid, as well as some other fatty acids (Additional file 1), were present in lower levels in the HCC tissues than in the adjacent noncancerous tissues (Fig. 1A).

### Alpha-linolenic acid inhibits HCC cell proliferation

**Fig. 2:**
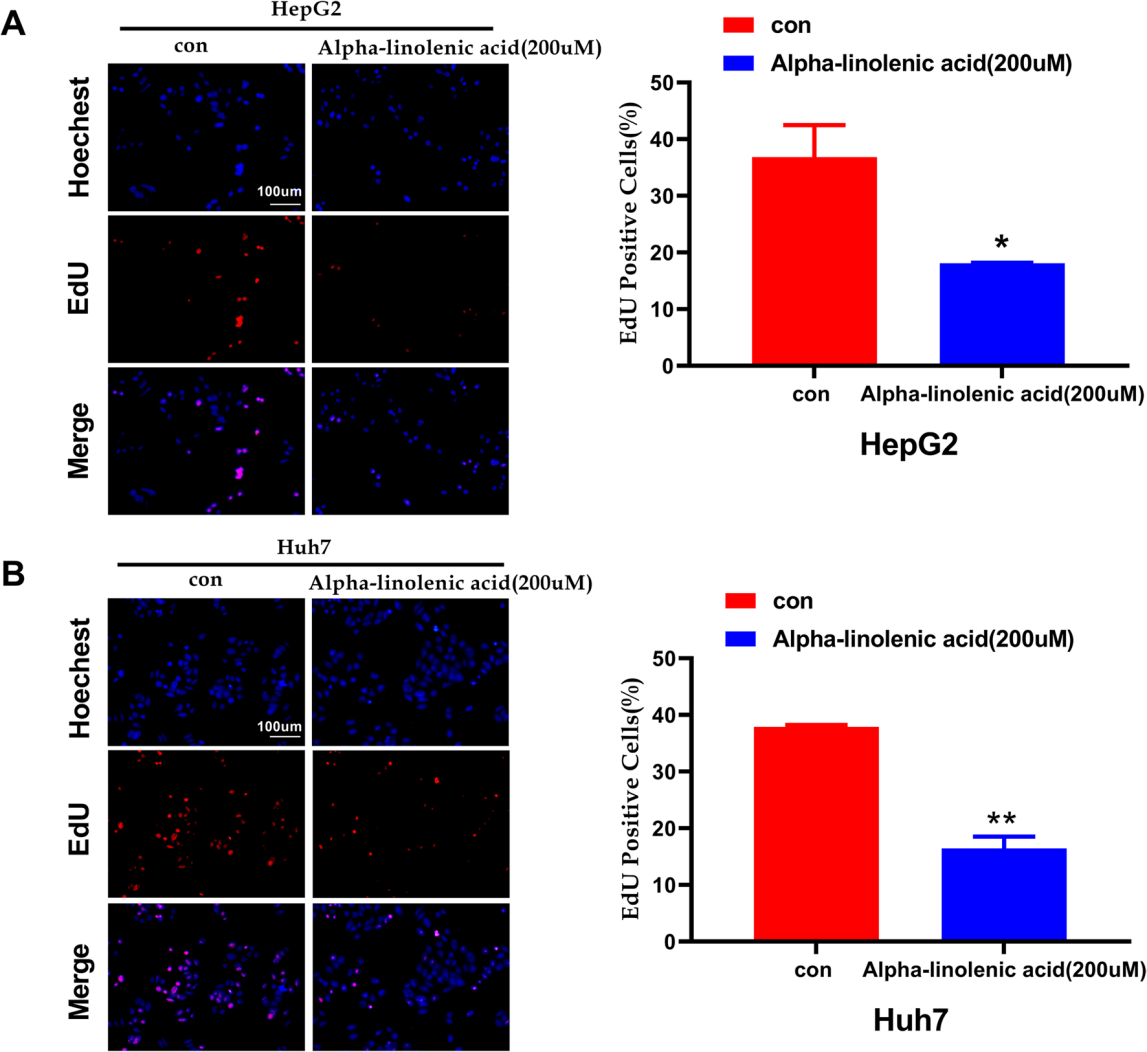
Alpha-linolenic acid inhibits HCC cell proliferation, as determined by the EdU assay. **A** EdU assay for assessing the proliferation of HepG2 cells. **B** EdU assay for assessing the proliferation of Huh7 cells. *p < 0.05, **p< 0.01

To understand the role of alpha-linolenic acid in proliferation, CCK-8 and EdU assays were adopted. The CCK-8 assay indicated that alpha-linolenic acid concentration (0, 100, 200 and 300 µM) inhibited HepG2 and Huh7 cell proliferation in a dose-dependent manner (Fig. 1C, D). To exclude the lipotoxic effect of alpha-linolenic acid accumulation in cells, normal liver cell line L02 cells were cultured with the same treatment conditions. Interestingly, the proliferation rate of L02 cells did not change when the cells were exposed to alpha-linolenic acid (Fig. 1B). The EdU assay showed that alpha-linolenic acid (200 µM) also significantly suppressed the proliferation of HepG2 and Huh7 cells (Fig. 2A, B). Thus, these results indicated that alpha-linolenic acid inhibited HCC cell proliferation in vitro.

### Alpha-linolenic acid inhibits HCC cell migration and invasion

**Fig. 3:**
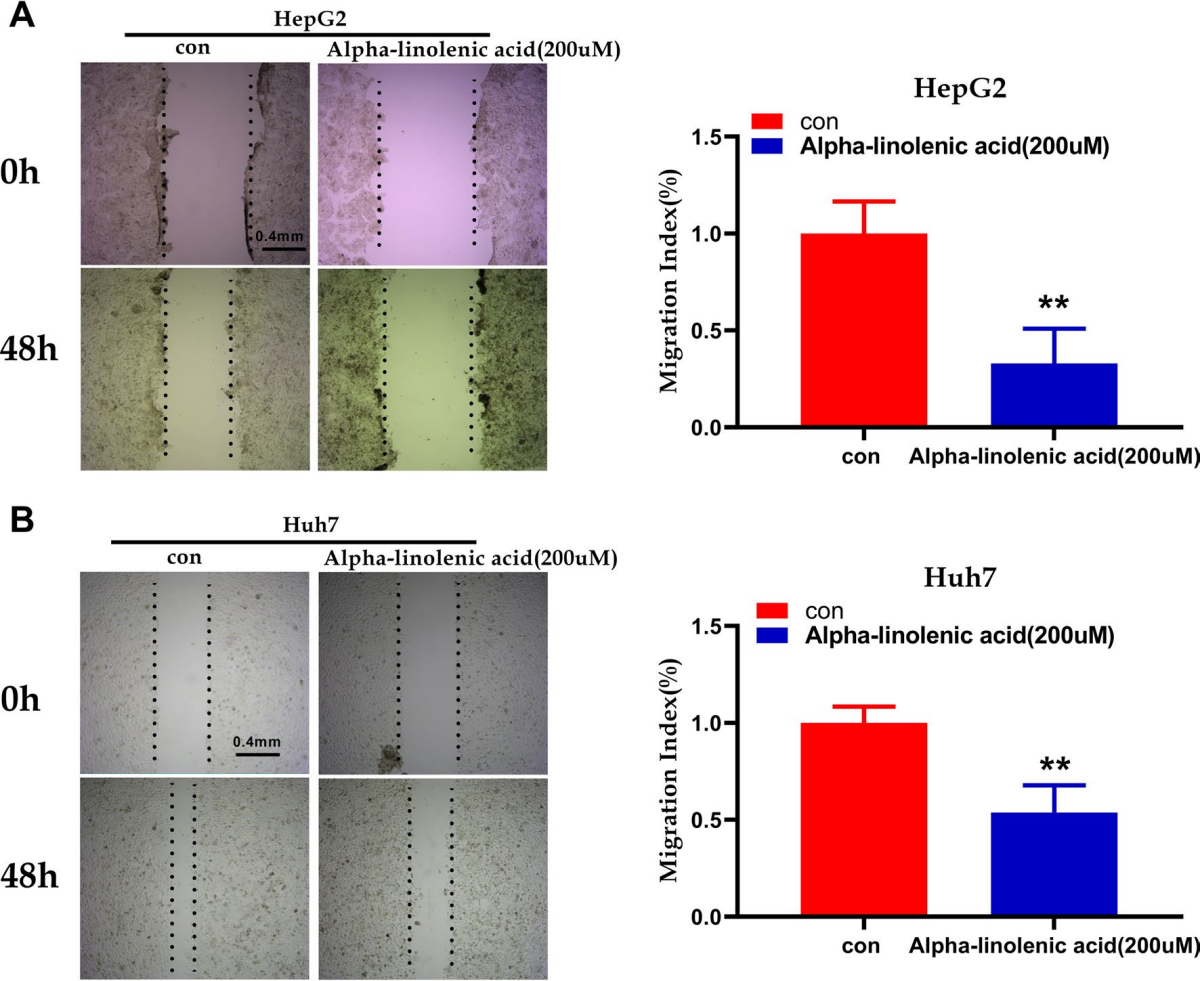
Alpha-linolenic acid inhibits the migration of HCC cells, as determined by wound healing assay. **A** The migratory property of HepG2 cells treated with alpha-linolenic acid (200 µM) was assessed with a wound healing assay. **B** The migratory property of Huh7 cells treated with alpha-linolenic acid (200 µM) was assessed with a wound healing assay. **p < 0.01

**Fig. 4:**
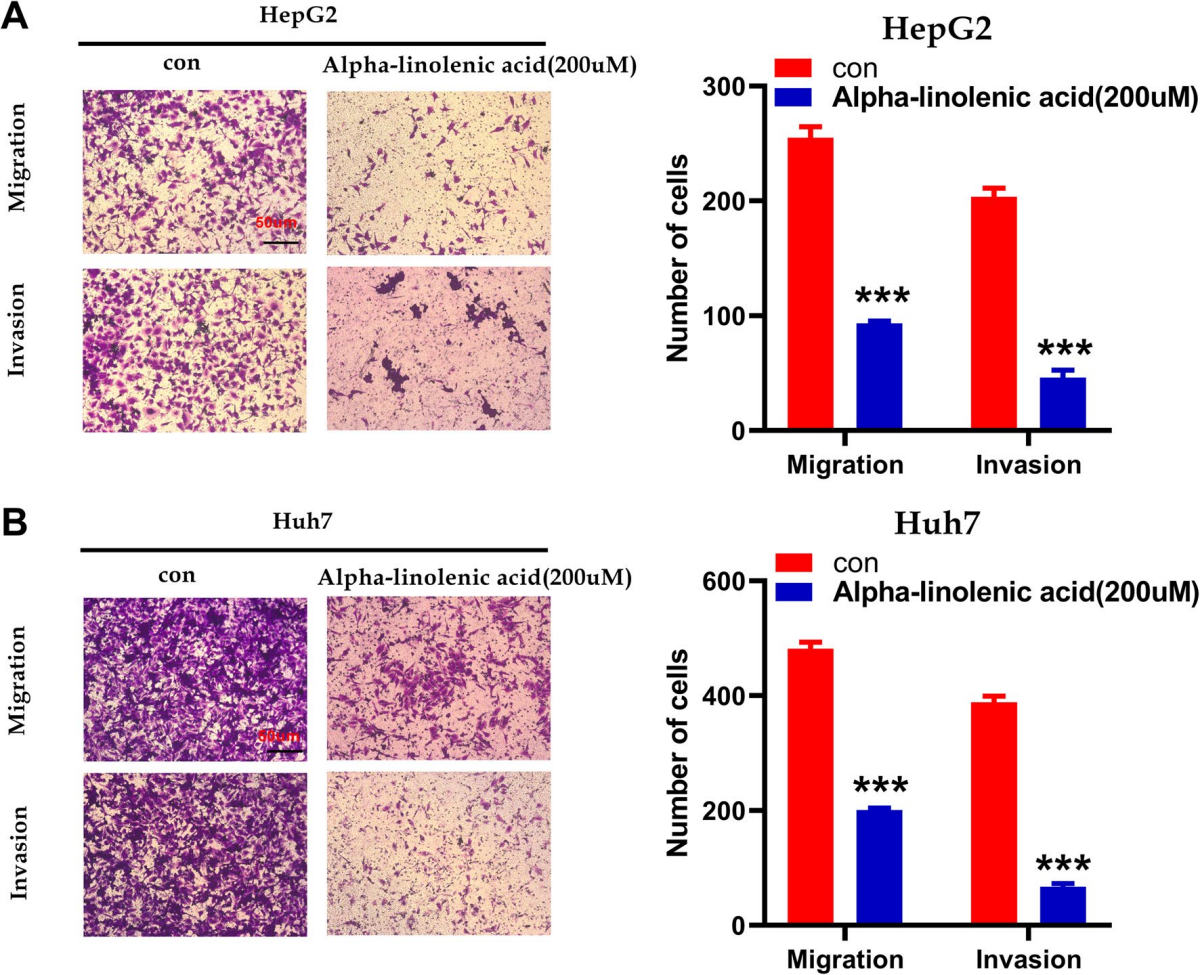
Alpha-linolenic acid inhibits the migration and invasion of HCC cells, as determined by Tranwell assay. **A** The migratory and invasive properties of HepG2 cells treated with alpha-linolenic acid (200 µM) were assessed with a Transwell assay. **B** The migratory and invasive properties of Huh7 cells treated with alpha-linolenic acid (200 µM) were assessed with a Transwell assay. ***p < 0.001

To explore the role of alpha-linolenic acid on the invasion and migration of HCC cells, Transwell migration and wound-healing assays were used. The heal areas were smaller in the alpha-linolenic acid group (200 µM) than in the control group in HepG2 and Huh7 cells (Fig. 3A, B). In addition, the Transwell assay demonstrated that alpha-linolenic acid group (200 µM) kept HepG2 and Huh7 cells from passing through the membrane (Fig. 4A, B). These findings indicated that alpha-linolenic acid repressed the migration and invasion of HCC cells.

### Alpha-linolenic acid suppresses the activity of the Wnt/β-catenin signaling pathway through activation of FXR and inhibits the expression of pro-oncogene c-myc

**Fig. 5:**
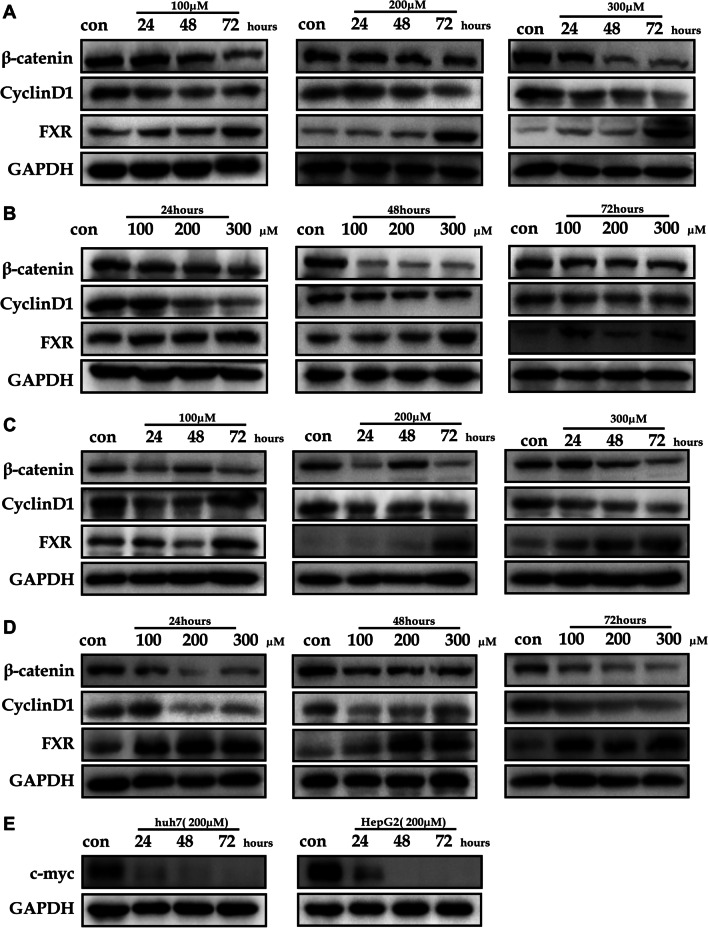
The expression of proteins in HCC cells treated with alpha-linolenic acid. **A** Alpha-linolenic acid promoted FXR expression and gradually decreased the expression of β-catenin and cyclin D1 in a time-dependent manner, and similar effects were observed at different doses (100 µM, 200 µM, and 300 µM) in HepG2 cells. **B** Alpha-linolenic acid promoted FXR expression and gradually decreased the expression of β-catenin and cyclin D1 in a dose-dependent manner, and similar effects were observed at different times(24, 48, and 72 h) in HepG2 cells. **C** Alpha-linolenic acid promoted FXR expression and gradually decreased the expression of β-catenin and cyclin D1 in a time-dependent manner, and similar results were observed at different doses (100 µM, 200 µM, and 300 µM) in Huh7 cells. **D** Alpha-linolenic acid promoted FXR expression and gradually decreased the expression of β-catenin and cyclin D1 in a dose-dependent manner, and similar results were observed at different times (24, 48, and 72 h) in Huh7 cells. **E** Alpha-linolenic acid inhibited the expression of pro-oncogene c-myc

The interaction between FXR and β-catenin impairs β-catenin/TCF4 complex formation to inhibit the progression of colon cancer [13]. Another study also demonstrated that FXR binds to β-catenin and suppresses its activity in HCC [14]. Thus, the effects of alpha-linolenic acid on the expression of FXR and the protein level of Wnt/β-catenin signaling pathway components were assessed in this study. In HepG2 cells, alpha-linolenic acid promoted the FXR expression, while the expression of β-catenin and its downstream target gene *cyclinD1* decreased in a time-dependent manner; these effects were dose (100 µM, 200 µM, and 300 µM) (Fig. 5A) and time(24, 48, and 72 h) dependent(Fig. 5B). Similar results were found in Huh7 cells (Fig. 5C, D). Furthermore, alpha-linolenic acid also inhibited the expression of pro-oncogene c-myc (Fig. 5E). Overall, alpha-linolenic acid promoted FXR expression to inactivate the Wnt/β-catenin signaling pathway in HCC.

### GW4064, the FXR agonist, suppresses HCC cells proliferation

**Fig. 6:**
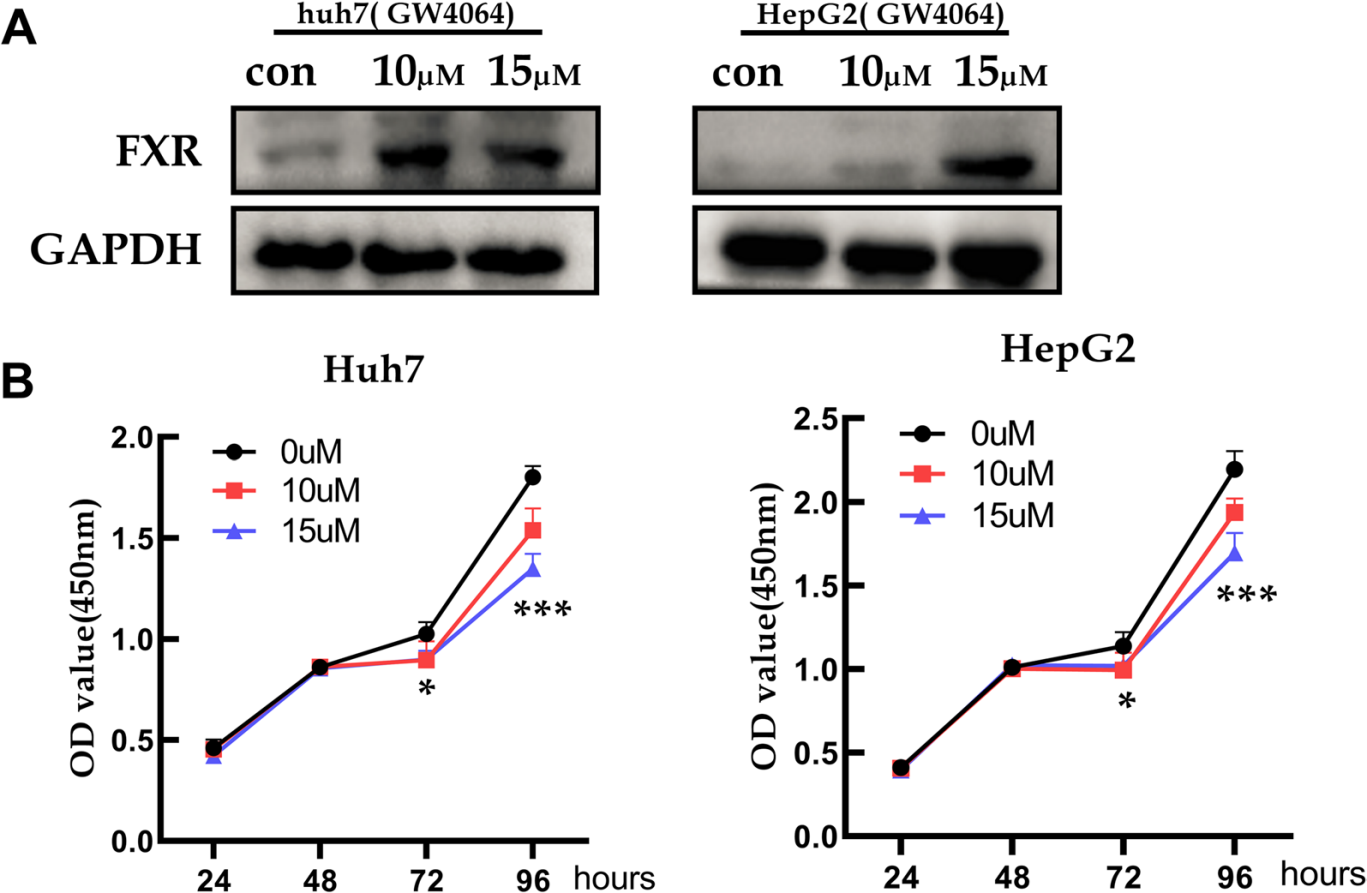
GW4064 suppressed HCC cell proliferation. **A** GW4064 increased the FXR expression verified by western blotting. **B** GW4064 inhibited the HCC cells proliferation by the CCK8 assay. *p < 0.05, ***p < 0.001

The HCC cells were treated with 10 µM or 15 µM GW4064, and the FXR overexpression was verified by western blotting (Fig. 6A). Then, the CCK-8 assay indicated that FXR overexpression inhibited HepG2 and Huh7 cell proliferation (Fig. 6B).

### HCC specimens with reduced FXR have elevated β-catenin expression

**Fig. 7:**
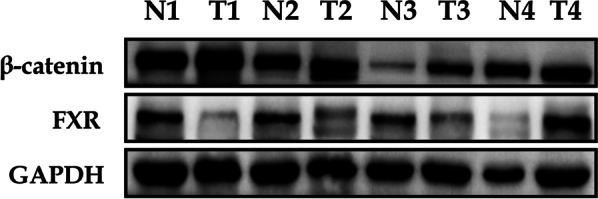
HCC specimens with reduced FXR have elevated β-catenin expression. Western blotting indicated HCC tissues with low expression of FXR had a high expression of β-catenin

In the previous cellular experiments, alpha-linolenic acid promoted FXR expression to inactivate the Wnt/β-catenin signaling pathway in HCC cells. Next, we also investigated the expression of FXR/β-catenin in the HCC tissues and adjacent noncancerous tissues. Western blotting indicated HCC tissues with low expression of FXR had a high expression of β-catenin (Fig. 7). These results showed that FXR was negatively correlated with β-catenin in human HCC specimens.

## Discussion

In the present study, it revealed that the alpha-linolenic acid level was lower in HCC tissues than in the adjacent noncancerous tissues, and alpha-linolenic acid suppressed the malignant potential of HCC cells. Assessment of the underlying mechanism revealed that alpha-linolenic acid enhances the expression of FXR, which suppresses the Wnt/β-catenin signaling pathway to impede HCC progression.

Abnormal lipid metabolism is important for tumour development. The total omega-6 polyunsaturated fatty acid (PUFA) level and omega-3 PUFA level are lower in colorectal cancer patients than in healthy people [15]. Linoleic acid and oleic acid levels are significantly lower in healthy men than in male CRC patients [16]. In a mouse model, lipid remodelling was involved in hepatocyte proliferation and HCC [17]. In the present study, it revealed that the content of alpha-linolenic acid was lower in HCC tissues than in nontumour tissues. Alpha-linolenic acid was found to have potential applications in breast cancer prevention and treatment [18, 19]. In human triple-negative breast cancer cells, alpha-linolenic acid inhibits migration by reducing twist1 expression, which promotes epithelial–mesenchymal transition [9]. Subsequently, we verified in this study that alpha-linolenic acid inhibits the malignant potential of HepG2 and Huh7 cells.

FXR, a nuclear receptor, suppresses bile acid synthesis and promotes bile acid enterohepatic circulation [20]. FXR is also a protective factor in HCC development. Su et al. [21] observed that downregulation of FXR was related to malignant clinicopathological characteristics in HCC and that FXR reduced the proliferation of HCC cells and inhibited tumour growth in nude mice. An FXR activator blocked nonalcoholic steatohepatitis-dependent HCC progression by suppressing the SOCS3/Jak2/STAT3 pathway [22]. The lack of FXR is likely associated with hepatocarcinogenesis in Abcb11-/-mice [23]. In HCC, FXR serves as a negative regulator through direct suppression of the Wnt/β-catenin pathway [14, 24]. In addition, FXR inhibits Wnt/β-catenin signaling in colorectal tumorigenesis [13]. Thus, FXR indeed plays an important negative function in the development of HCC. Wnt/β-catenin signaling is activated in different tumours [25]. The Wnt/β-catenin pathway is mostly inactive in the mature healthy liver, however, Wnt/β-catenin signaling is frequently activated and facilitates tumour development in HCC [26]. Wnt/β-catenin signaling-related targets, such as c-myc and cyclin D1, are critical contributors to tumour cell proliferation potential [27]. Therefore, FXR/Wnt/β-catenin/cyclin D1 signaling as the target pathway for alpha-linolenic acid was chosen in this study. In the present study, it revealed the inhibitory effect of alpha-linoleic acid on malignant potential of HCC cells. HCC cells were treated with alpha-linolenic acid and it revealed that alpha-linolenic acid increased FXR expression and gradually decreased the expression of β-catenin and its downstream target gene cyclin D1, meanwhile, alpha-linolenic acid also reduced the expression of pro-oncogene c-myc. These findings suggest that FXR/Wnt/β-catenin is a pathway through which alpha-linolenic acid inhibits HCC development.

## Conclusion

The data revealed that the content of alpha-linolenic acid was reduced in HCC tissues and showed that alpha-linolenic acid suppressed HCC development through the FXR/Wnt/β-catenin signaling pathway. These novel findings have identified a previously unrecognized relationship between alpha-linolenic acid and FXR/Wnt/β-catenin signaling, thus providing a novel opportunity for HCC intervention. In addition, rational use of alpha-linolenic acid may prevent the occurrence of liver cancer in the future.

## Supplementary Information

**Additional file 1**. Fatty acids profile between HCC tissues and corresponding adjacent noncancerous tissues with GC/MS analysis.

## Data Availability

The data described in this manuscript are contained in published articles or the experiment data are available upon reasonable request.

## Abbreviations

HCC: Hepatocellular carcinoma
GC/MS: Gas chromatography/mass spectrometer
CCK-8: Cell Counting Kit-8
FXR: Farnesoid X receptor
